# Unlocking Quantum Catalysis in Topological Trivial Materials: A Case Study of Janus Monolayer MoSMg

**DOI:** 10.1002/smsc.202400160

**Published:** 2024-07-22

**Authors:** Ying Yang, Jialin Gong, Xiaotian Wang, Zhenxiang Cheng, Tie Yang

**Affiliations:** ^1^ College of Physics and Electronic Engineering Chongqing Normal University Chongqing 401331 China; ^2^ School of Physical Science and Technology Southwest University Chongqing 400715 China; ^3^ Institute for Superconducting and Electronic Materials Faculty of Engineering and Information Sciences University of Wollongong Wollongong 2500 Australia

**Keywords:** Gibbs free energies, hydrogen‐evolution reaction catalysts, obstructed atomic insulators, obstructed edge states, obstructed Wannier charge centers

## Abstract

The emerging field of topological catalysis has received significant attention due to its potential for high‐performance catalytic activity in the hydrogen‐evolution reaction (HER). While topological materials often possess fragile surface states, trivial topological materials not only offer a larger pool of candidates but also demonstrate robust surface states. As a result, the search for topological catalysts has expanded to include trivial schemes. In this study, a novel 2D Janus monolayer, MoSMg, which demonstrates exceptional obstructed atomic insulating behavior, is presented. Crucially, this trivial metallic topological state exhibits clean obstructed surface states, leading to a significant enhancement in catalytic performance for the HER in electrochemical processes, particularly under high hydrogen coverage. Moreover, the edge sites of this MoSMg monolayer exhibit even more superior catalytic activity, characterized by near‐zero Gibbs free energies. In these findings, the way is paved for exploring new avenues in the design of quantum electrocatalysts, especially within the realm of trivial topological materials.

## Graphical abstract

The search for topological catalysts has expanded to include trivial materials. A novel Janus monolayer, MoSMg, demonstrates exceptional obstructed atomic insulating behavior with clean obstructed surface states, leading to a significant enhancement in catalytic performance for the hydrogen evolution reaction.

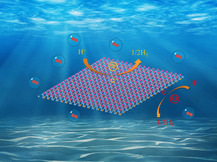

## 1 Introduction

The hydrogen‐evolution reaction (HER), represented by the equation ${\text{2H}}^{+}+{\text{2e}}^{-}\to H_{2}$, plays a crucial role in the process of water splitting.^[^ ^1^ ^]^ However, a high‐performance electrocatalyst is required to expedite this process. Currently, platinum (Pt) is extensively employed as a catalyst.^[^ ^2^, ^3^, ^4^ ^]^ However, its elevated cost and limited availability have prompted researchers to investigate alternative noble metal catalysts for the HER that are both cost‐effective and capable of maintaining comparable HER activity to Pt.^[^ ^5^, ^6^, ^7^ ^]^ This exploration involves a multifaceted approach that includes not only the identification of alternative noble and non‐noble metals but also the engineering of catalysts at the nanoscale to enhance their activity and stability.^[^ ^8^, ^9^, ^10^, ^11^, ^12^, ^13^, ^14^, ^15^, ^16^ ^]^ Advances in computational chemistry and materials science are playing a crucial role in the search for alternative catalysts for HER.^[^ ^17^ ^]^ Theoretical models and simulations can predict the catalytic activity of materials before they are synthesized, guiding the experimental efforts toward the most promising candidates.^[^ ^18^, ^19^, ^20^, ^21^ ^]^ Computational studies can also reveal the fundamental mechanisms at play in HER, providing insights into how catalysts can be further optimized.^[^ ^22^, ^23^, ^24^ ^]^ The development of alternative catalysts for HER represents a dynamic and interdisciplinary field of research. Through the combination of experimental and computational approaches, scientists are making significant progress in identifying and optimizing materials that could potentially replace platinum in water splitting applications, paving the way for more sustainable and cost‐effective hydrogen production.

The discovery of topological materials has significantly expanded the potential for the advancement of high‐performance electrocatalysts.^[^ ^25^, ^26^, ^27^, ^28^, ^29^, ^30^, ^31^, ^32^, ^33^, ^34^, ^35^, ^36^, ^37^, ^38^, ^39^ ^]^ The first emphasis in the development of high‐performance quantum electrocatalysts was placed on topological insulators, a specific category of topological materials distinguished by their insulating behavior for the bulk while exhibiting robust conducting states on the edge or surface. For example, Chen et al. provided evidence that the robust topological surface state (TSS) in 3D topological insulators can significantly promote surface reactions through its ability to operate as an efficient electron bath.^[^ ^40^ ^]^ Therefore, the proposition of a TSS serving as an electron bath can potentially introduce novel design ideas that extend beyond the traditional d‐band theory in the field of heterogeneous catalysis.^[^ ^17^ ^]^ Further research has been conducted on high‐performance quantum electrocatalysts, expanding the scope of exploration from topological insulators to include topological semimetals. A series of topological semimetals, including Weyl semimetals, multifold nodal‐point semimetals, and nodal line semimetals, have been considered to be high‐performance quantum electrocatalysts toward HER. The relationship between the Gibbs free energy (Δ*G*_H*_) and the density of TSSs at the Fermi level in the majority of predicted materials candidates is strongly established.^[^ ^22^ ^]^ This correlation offers direct support for the notion that TSSs can serve as a universal descriptor for catalytic activity in topological catalysis.

Many materials are currently predicted as topological insulators or topological semimetals with nontrivial boundary states, which may boost catalysis. However, the number of topological insulators/semimetals is still quite tiny in comparison to the topological trivial materials, especially *topological trivial insulators*. In the topological quantum chemistry theory, a topological trivial insulator is a material whose band representations (BRs) of valence bands can be characterized by a sum of elementary BRs (EBRs), which means that the trivial state can be described by a set of exponentially localized Wannier functions. In general, trivial insulators can be classified into two categories: obstructed atomic insulators (OAIs) and atomic insulators.^[^ ^41^, ^42^, ^43^ ^]^ This categorization is based on the Wyckoff positions (WPs) where the orbitals responsible for inducing the BRs are situated. For the atomic insulators, their BRs are induced from the orbitals that are located at occupied WPs; whereas, for the OAIs, their BRs are induced from the orbitals centered at the occupied WPs and the orbitals located at empty WPs (atom‐unoccupied WPs). Such atom‐unoccupied WPs in OAIs are named obstructed Wannier charge centers (OWCCs). When the cleavage termination cuts through those OWCCs in an OAI, the metallic obstructed surface states (OSSs) will occur.^[^ ^44^ ^]^ Unlike TSSs, the OSSs are clean, located between the large energy gap, and simply to distinguish in the energy spectrum from the bulk states, which is advantageous for theoretical analysis and experimental detection.^[^ ^45^ ^]^ Remarkably, it has been suggested that the OSSs in an OAI can be used as a descriptor to define catalytic active sites in 3D inorganic crystalline materials.^[^ ^29^ ^]^ That is, the crystal surface with OSSs could be active for molecules bonding and adsorption. It is important to keep in mind that all of the proposed quantum catalysts with OSSs are based on 3D bulk materials. When compared to 3D materials, 2D materials with abundant active sites in the form of surface atoms and edge sites and superior electrical conductivity along 2D conducting channels are potential prospects for high‐performance electrocatalysts.^[^ ^46^, ^47^, ^48^, ^49^, ^50^, ^51^, ^52^, ^53^, ^54^, ^55^ ^]^ In light of this, the development and design of 2D quantum catalysts with metallic obstructed boundary states and high catalytic performance should be continued, and additional advancements in specific fields are anticipated.

In this study, we have developed a novel 2D material MoSMg by replacing one layer of S with a layer of Mg in the monolayer MoS_2_. This substitution results in a Janus structure due to the different atomic radius and electronegativity. We would like to clarify that we also considered substitutions with other alkaline‐earth‐metal elements. However, these resulting structures were found to be dynamically unstable and were thus excluded from our study. In addition to its exceptional stability, the Janus monolayer MoSMg also demonstrates OAI behavior. Importantly, this unique structure features clean OSSs and, remarkably, metallic topological trivial states that greatly enhance its catalytic performance for HER in electrochemical processes. Furthermore, we investigated the performance of the obstructed OWCC site under various hydrogen coverage conditions. The OWCC site consistently displayed robust HER performance, particularly at high hydrogen coverage. We also examined the edge sites of the monolayer MoSMg, which exhibited even more superior HER catalytic activity. Specifically, the armchair edge sites demonstrated near‐zero Gibbs free energies, indicating highly favorable HER kinetics. The combination of OAI behavior and exceptional HER catalytic activity represents a novel finding in the field. By identifying this 2D Janus MoSMg material, our study opens up new possibilities for designing quantum electrocatalysts with desirable performance characteristics.

## 2 Computational Methodology

First‐principles calculations based on density‐functional theory (DFT) were performed using the Vienna Ab initio Simulation Package (VASP).^[^ ^56^, ^57^ ^]^ The Perdew–Burke–Ernzerhof functional,^[^ ^58^ ^]^ within the generalized gradient approximation,^[^ ^59^ ^]^ was employed for the exchange–correlation potential. A cutoff energy of 400 eV was utilized. The Brillouin zone was sampled using a Monkhorst–Pack k‐mesh with dimensions of 9 × 9 × 1. During lattice optimization, energy and force convergence criteria were set at 10^−6^ eV and 0.01 eV Å^−1^, respectively. To account for long‐range van der Waals interactions, the DFT‐D2 method was employed,^[^ ^60^ ^]^ and a vacuum layer with a thickness of 30 Å was included to prevent artificial interactions between periodic images. To assess dynamic stability, phonon spectra were calculated using density‐functional perturbation theory implemented in the PHONOPY program.^[^ ^61^, ^62^, ^63^ ^]^ Additionally, thermal stability was evaluated through ab initio molecular dynamics (AIMD) simulations in the canonical ensemble,^[^ ^64^, ^65^ ^]^ conducted on a 5 × 5 × 1 supercell. The electronic band structures were computed considering the spin–orbit coupling effect.^[^ ^66^ ^]^ The topological properties were determined using the IRVSP code based on the topological quantum chemistry (TQC) theory,^[^ ^67^, ^68^, ^69^, ^70^ ^]^ while the edge states were calculated using Wannier functions and the WannierTools package.^[^ ^71^, ^72^, ^73^, ^74^ ^]^ Furthermore, for the evaluation of catalytic properties, the Gibbs free energy $G_{H^{*}}$ (where * denotes an active site on the surface and H^∗^ represents the reaction intermediate) was computed using the method proposed by Norskov et al.^[^ ^75^, ^76^, ^77^ ^]^

## 3 Results and Discussion

### 3.1 Structure and Stability

**Figure 1:**
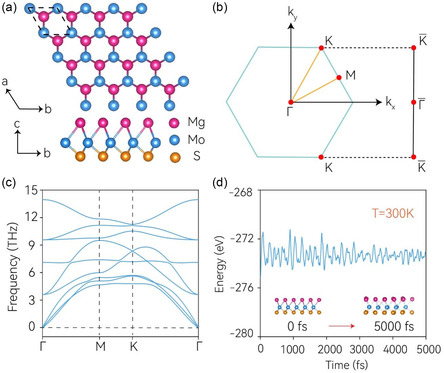
a) Top and side views of the Janus monolayer MoSMg, with the unit cell indicated by black dashed lines. b) The 2D Brillouin zone and its projection onto the [010] edge. c) Phonon dispersion calculated for the MoSMg monolayer. Nine phononic bands corresponding three atoms in the unit cell. d) Ab initio molecular dynamics simulation of MoSMg at 300 K using a 5 × 5 × 1 supercell configuration. A slight thermal‐induced structural fluctuation can be seen from the initial and final lattice captures.

The monolayer MoSMg exhibits a layered structure with three planar atomic layers stacked together, as shown in **Figure** **1**a, which corresponds to the typical Janus structure, and its hexagonal structural symmetry with space group P3m1 forms two sorts of honeycomb lattices vertically piled up, in which each Mo atom is bonded with three Mg/S atoms. This Janus monolayer MoSMg can be obtained by substituting one layer of S atoms with Mg atoms in the MoS_2_ monolayer and thus the hitherto layer distribution in the monolayer MoSMg comes from the different S and Mg atoms in terms of both atomic size and electronic configurations. The unit cell is indicated by the black dashed lines in the top view of Figure 1a, and it has a single formula MoSMg. Based on the fully relaxed structure, the obtained lattice parameters are *a* = *b* = 3.041 Å, and more detailed atomic positions and bond length distributions are provided in Table S1, Supporting Information. Before delving into the intricacies of the electronic properties of the lattice, it is of paramount importance to ascertain its dynamical and thermal stabilities, which are also very important key factors for the HER application. To achieve this, we employed density‐functional perturbation theory to acquire the force constants, utilizing the VASP for implementation. Following this, we employed the PHONOPY package to compute the phonon‐dispersion spectrum. As evidenced in Figure 1c, the absence of imaginary frequencies in the phonon spectrum corroborates the dynamic stability of the Janus monolayer MoSMg. To further assess its thermal stability, especially for room temperature application, we conducted AIMD calculations within a 5 × 5 × 1 supercell framework. Figure 1d reveals that after executing 5000 fs at a temperature of 300 K, the final states only manifested slight thermal‐induced fluctuations. Notably, these fluctuations occurred without any bond dissociation or geometric reconfigurations, thereby solidifying the assertion that Janus monolayer MoSMg maintains thermal stability at ambient conditions. The MoSMg monolayer, though not yet realized in a practical setting, presents a compelling subject for future synthesis. A promising avenue for its creation could involve the doping of Mg atoms into the MoS_2_ lattice via molecular beam epitaxy. This approach mirrors the successful doping of C atoms into phosphorene, resulting in the formation of phosphorene carbide.^[^ ^78^ ^]^ This proposed method, while theoretical at this stage, provides a promising foundation for future experimental endeavors in the synthesis of the MoSMg monolayer. Considering the high oxidization tendency of MoS_2_, we have also assessed the stability of the Janus MoSMg monolayer against oxidation and detailed information can be found in Supporting Information. It is found that MoSMg, similar to MoS_2_, is prone to oxidation. To provide a practical strategy, a carbon shell protection method has been demonstrated to be an effective approach to mitigate the oxidation for MoS_2_,^[^ ^79^ ^]^ and thus we anticipate that similar protective measures could be applied to the MoSMg monolayer, potentially preserving its structural integrity and electronic properties.

### 3.2 OAI State and HER Activity

**Figure 2:**
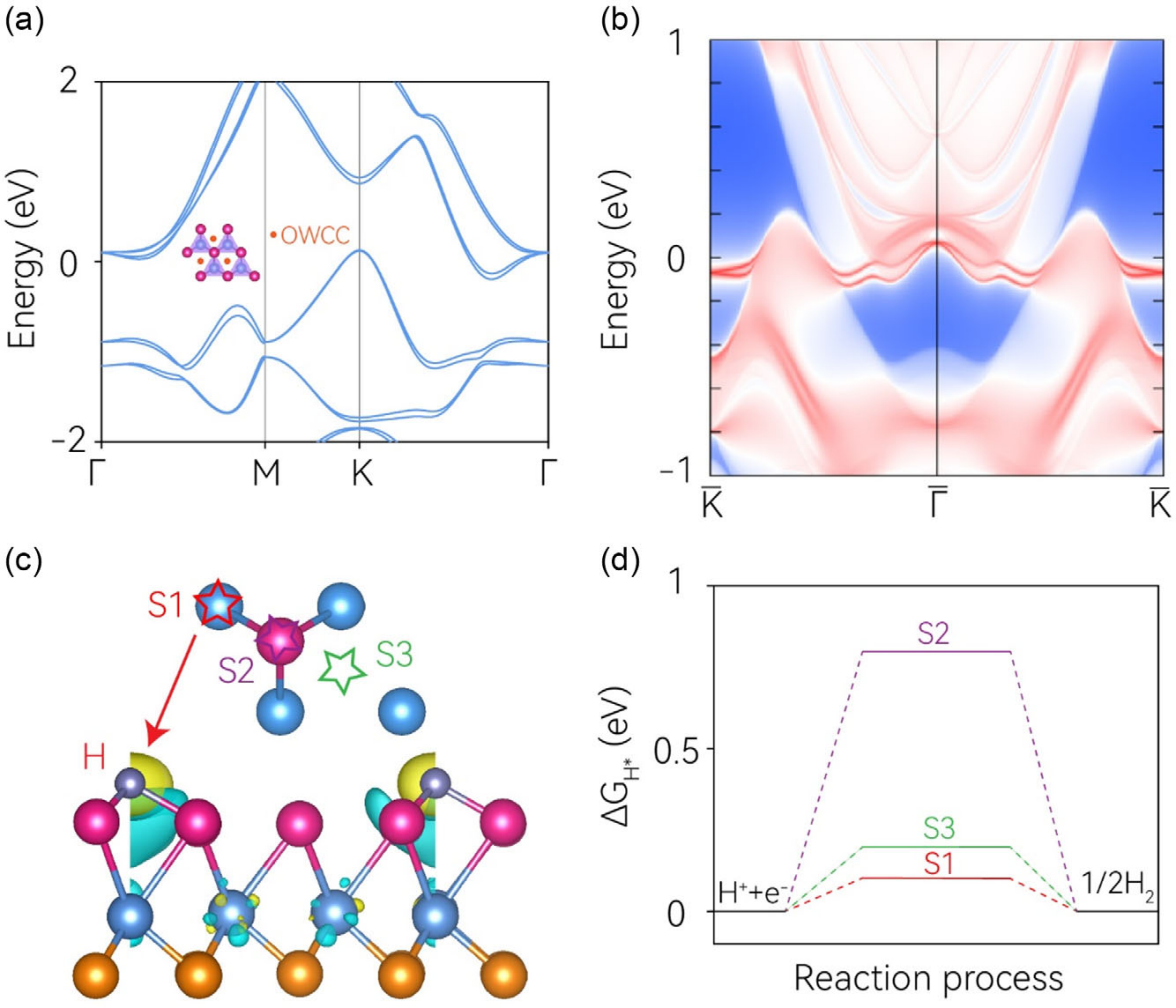
a) The electronic band structure of the Janus monolayer MoSMg with the consideration of the spin–orbital coupling effect. Inset illustrates the distribution and location of the OWCCs. b) Edge state for the [010] edge projection with the metallic obstructed edge state. c) Three hydrogen adsorption sites on the MoSMg monolayer and the charge‐transfer visualization at S1 site. d) Calculated Δ*G*_H*_ for hydrogen adsorption at different sites.

Based on the obtained lattice structure, the electronic band structure of the monolayer MoSMg has been calculated and reported in **Figure** **2**a. In consideration of the involvement of the heavy transition metal element Mo, the spin–orbital coupling effect has been taken into account. The monolayer MoSMg exhibits indirect band configuration, that is, the conduction band maximum (CBM) occurs at the high symmetry point K whereas the valence band minimum (VBM) is located along the high symmetry path K–Γ. Since the energies of both CBM and VBM overlap with the Fermi energy level, this 2D material exhibit metallic behavior. Moreover, further symmetry analysis reveals that its band structures are topologically trivial, especially under the consideration of spin–orbital coupling effect.

In the context of topological trivial insulators, their populated electronic bands can be represented by nonnegative integer linear combinations of EBRs (LCEBRs) as per the TQC theory.^[^ ^68^, ^69^, ^70^ ^]^ Given that the MoSMg monolayer is a 2D material, we only consider the BRs of high symmetry points Γ, M, and K. The EBRs can be derived from the combinations of EBRs of maximal WPs 1a, 1b, and 1c. Consequently, we select the maximal WPs 1a, 1b, and 1c for EBR decomposition.^[^ ^45^ ^]^ The resulting LCEBRs of the populated electronic bands of MoSMg are presented in Table S2, Supporting Information. Intriguingly, for MoSMg, all the LCEBRs reveal three EBRs (E1@1a, E1@1b, and E1@1c) with a nonzero integer combination. This implies that these three EBRs are indivisible and must be linked to the electron‐filled WPs by 1a, 1b, and 1c. The Mo atom occupies the 1a site and the Mg and S atoms take the 1b sites, while the 1c site remains unoccupied. As per the OAI definition, MoSMg qualifies as an OAI, and the 1c site is the OWCCs. A critical aspect of the OAI characteristic involves verifying whether the unoccupied WPs possess localized charges, i.e., electron filling. To better understand the real‐space charge localizations of MoSMg, we visualized the electron localization function on the central Mo‐atom layer, as shown in the inset of Figure 2a. It is evident that the charges localize primarily at the atom‐unoccupied 1a WP. Similar to the TSSs in topological insulators, the OSSs can be present when the cleavage terminations cut exactly through the OWCCs in OAIs. This can be confirmed by our surface states calculation on the (10) edge, see Figure 2b, in which multiple metallic boundary states can be observed and they distribute between the CBM and VBM. Note that these metallic boundary states can behave like high conduction channel and serve as efficient electron source, especially when they are located at the Fermi energy. However, a distinguishing feature of OSSs from TSSs is the fact that OSSs are active site related and they can only occur at some specific termination surfaces. This is the reason why OSSs have been successfully selected as a straightforward descriptor for the prediction and determination of catalytic active state.

Given that both the metallic bulk and edge states can intrinsically offer high conductivity, a property that is highly beneficial for catalytic activities, we further delve into the HER performance of the monolayer MoSMg. We focus on three specific sites: the two top sites, S1 and S2, and the OWCC site, S3, as illustrated in Figure 2c. After relaxation optimization, we find that all three of these sites can effectively stabilize hydrogen atoms. To show the charge transfer during catalysis, we have calculated the charge‐density difference as an example on the S1 site, as shown in Figure 2c. The charge depletion of the MoSMg monolayer is observed as the yellow region around the top layer while charge accumulation occurs as the blue region on the H atom. The hydrogen adsorption free energy (Δ*G*_H*_), a critical parameter in evaluating the HER performance, is computed for these three sites by using the computational hydrogen electrode method, initially developed by Norskov et al. The results of this calculation are reported in Figure 2d and detailed Δ*G*_H*_ values are documented in Table S3, Supporting Information. Notably, the Δ*G*_H*_ value at the Mo top S1 site can be as low as 0.103 eV, indicating exceptional electrocatalytic performance for the HER. This suggests that the S1 site could potentially be a highly efficient catalyst for hydrogen production in electrochemical water splitting, a critical process in sustainable hydrogen production. In addition, the calculated Δ*G*_H*_ value for the OWCC site S3 is 0.180 eV, suggesting that this site also possesses some degree of HER activity. This indicates that the S3 site, while not as active as the S1 site, can still contribute to the overall HER performance of the MoSMg monolayer. In contrast, the Mg top site S2 has a Δ*G*_H*_ value of 0.797 eV, which is significantly far from zero and thus indicates inert HER activity. This suggests that the S2 site is likely not a significant contributor to the catalytic activity of the MoSMg monolayer. From these observations, it is clear that the S1 and S3 site can be identified as the active site for HER in the MoSMg monolayer. This understanding of the site‐specific catalytic activities can provide valuable insights for the design and optimization of 2D material‐based catalysts for electrochemical hydrogen production.

**Figure 3:**
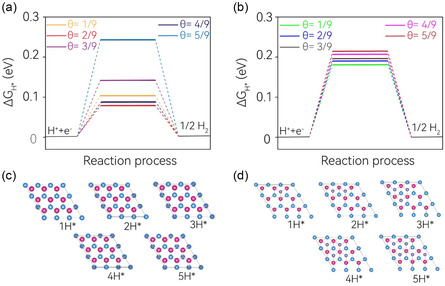
a,b) Calculated Δ*G*_H*_ with H coverage on the MoSMg monolayer for S1 and S3 sites, respectively. c,d) The corresponding atomic structure of hydrogen adsorption on the S1 and S3 sites of the 3 × 3 MoSMg monolayer surface.

In addition to the impact of sparse hydrogen adsorption on HER, the degree of hydrogen coverage can significantly influence the catalytic activity toward HER. The performance of HER is primarily dictated by the nth hydrogen adsorbate with a Δ*G*_nH*_ value closest to thermoneutral, ≈0 eV. To explore the effects of hydrogen coverage, we employed a 3 × 3 supercell. The calculated hydrogen coverage‐dependent Δ*G*_H*_ is presented in **Figure** **3**, and the detailed Δ*G*_H*_ values are documented in Table S4, Supporting Information. We examined only two sites: the top Mo site S1 and the OWCC site S3. The hydrogen coverage ratio (denoted as “*θ*”) considered ranged from 1/9 to 5/9. With an increase in hydrogen coverage, Δ*G*_H*_ displays a pronounced coverage‐dependent behavior at the S1 site. At low “*θ*”, Δ*G*_H*_ exhibits slight fluctuations, and a minor increase in coverage further reduces Δ*G*_H*_, indicating enhanced catalytic activity at “*θ*” of 2/9 and 3/9. However, with further increases in hydrogen coverage, Δ*G*_H*_ experiences a substantial rise, particularly a sudden leap to 0.242 eV at “*θ*” of 5/9. This suggests a significant deterioration in the corresponding HER performance. Contrastingly, the OWCC site presents a distinctly different scenario regarding the hydrogen coverage effect. As “*θ*” increases, Δ*G*_H*_ gradually rises, but the variation is considerably smaller, indicating robust and consistent HER performance. This stability is highly desirable and advantageous for HER applications under practical conditions. At the highest “*θ*” of 5/9, Δ*G*_H*_ only increases from 0.180 eV at “*θ*” of 1/9 to 0.215 eV. Interestingly, this value is lower than that at the S1 site under the same hydrogen coverage. Consequently, the OWCC site not only maintains consistent HER activity under varying hydrogen coverage conditions but also exhibits superior HER performance at high hydrogen coverage. This suggests that the OWCC site could be a promising catalyst for HER in practical applications. These findings provide important insights into the design and optimization of catalysts for efficient and sustainable hydrogen production. It should be noted that this non‐monotonous behavior of the calculated Δ*G*_H*_ values with hydrogen coverage has been previously reported^[^ ^80^, ^81^ ^]^ and is highly material‐dependent and can vary significantly case by case.

### 3.3 Nanoribbon HER Activity

**Figure 4:**
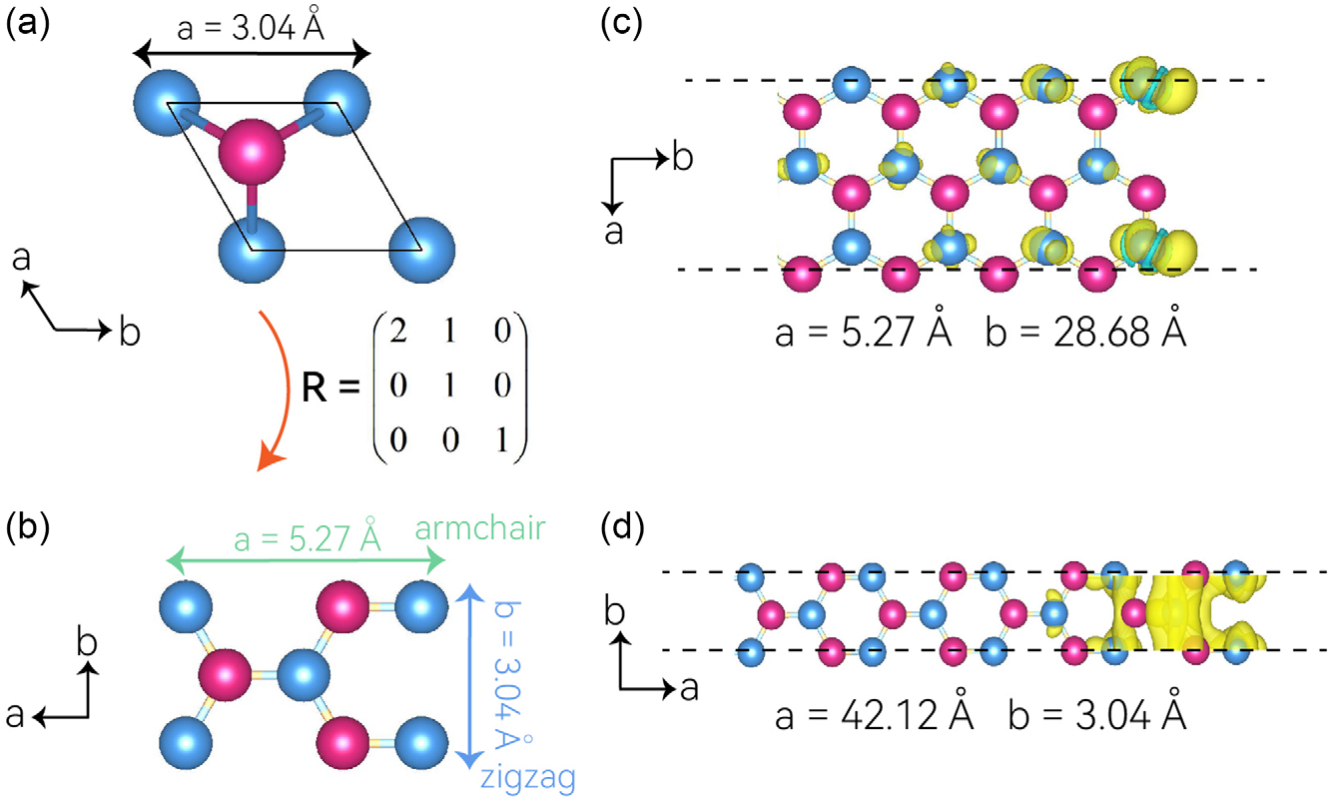
a) The hexagonal unit cell of Janus monolayer MoSMg with the lattice constant indicated. b) The transformed orthorhombic unit cell, which is obtained by using the rotation transformation matrix (R) presented. Two different border terminations can be derived, namely, armchair and zigzag. c,d) Zigzag and armchair terminations of the nanoribbons (along with lattice parameters) and their corresponding edge Fermionic accumulations.

Beyond the basal sites of the MoSMg monolayer, edge sites also demonstrate potential for catalytic HER activities. To investigate the heterogeneous catalysis at these edge sites, we transitioned from the original hexagonal basis vector to an orthorhombic basis vector via basis vector rotation. Both the original and transformed unit cells are depicted in **Figure** **4**a,b, with the applied rotation matrix (R) also provided for reference. The resultant orthorhombic lattice structure displays two distinct edge terminations: zigzag and armchair, with corresponding lattice constants of 3.04 Å along the zigzag edge and 5.27 Å along the armchair edge, as illustrated in Figure 4b. High electron accumulation at the edge sites, akin to the high conductivity required for superior HER performance, is of significant importance. This is because it can supply both a sufficient electron source and a high charge‐transfer path, which are crucial for catalytic activity. The computed electron localization functions at the two edge terminations are presented in Figure 4c,d. It can be distinctly observed that both armchair and zigzag edge sites exhibit strong and robust Fermionic accumulations. This strong electron accumulation at the edge sites suggests a high potential for HER catalytic activity along these two edges. Therefore, these results highlight the importance of edge sites in 2D materials like MoSMg in enhancing catalytic activities, providing a new perspective in the design and optimization of 2D catalytic materials for HER.

**Figure 5:**
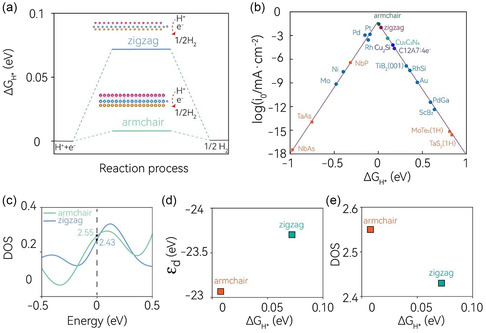
a) Calculated Δ*G*_H*_ for hydrogen adsorption at two edge sites of the monolayer MoSMg. The two insets illustrate the zigzag and armchair edge sites with the hydrogen stabilized at the Mo positions. b) Volcano plot for Δ*G*_H*_ of the two edge sites with that of various pure noble metals, topological Weyl semimetals, and other experimentally synthesized 2D monolayer materials and typical topological catalysts. The data are taken from the literature.^[^ ^18^, ^22^, ^30^, ^31^, ^32^, ^75^, ^84^, ^85^, ^86^, ^87^ ^]^ c) The edge DOSs around the Fermi level for the two edge sites. d,e) Plot of the d‐band center (*ε*_d_) and edge DOSs as a function of Δ*G*_H*_ for the two edge sites.

Following the same procedure as for the basal sites, we proceeded to calculate the Δ*G*_H*_ values of the two edge sites. The results of these calculations are presented in **Figure** **5** and detailed Δ*G*_H*_ values are reported in Table S5, Supporting Information. After substantial structural relaxation, hydrogen atoms can be stabilized at the Mo site of both armchair and zigzag edges, which are depicted in the two insets in Figure 5a. The Δ*G*_H*_ values for these edge sites are markedly smaller than those of the previously examined basal sites, with 0.075 eV for the zigzag edge and 0.001 eV for the armchair edge. These near‐zero Δ*G*_H*_ values for the edge sites suggest an even higher level of HER activity, outperforming the basal sites. To further illustrate this, we plotted a volcano curve of the exchange current density against the corresponding Δ*G*_H*_ value in Figure 5b. For a more comprehensive comparison, we included data from Pt‐group noble metals, several topological Weyl metals/semimetals, and other experimentally synthesized 2D monolayer materials. Notably, the two edge sites are positioned at the peak of the HER volcano curve, indicating optimal catalytic performance. In fact, these edge sites even surpass the catalytic performance of the state‐of‐the‐art nodal metal Pt. This finding underscores the potential of these edge sites in monolayer MoSMg as highly efficient catalysts for HER, potentially outperforming traditional noble metal catalysts.

To delve deeper into the exceptional HER catalytic performance of the two edge sites, we computed their surface density of states (DOSs) around the Fermi level, as depicted in Figure 5c. It is worth noting that in traditional electrocatalysts, surface‐dependent catalytic activities are commonly observed. The underlying mechanism is typically associated with the positions of d‐band centers and the specific surface chemical environments. To decode the differential catalytic activities of the two edge sites in the MoSMg monolayer, we examined the variations in surface DOSs and d‐band center (*ε*_d_) on different edge sites relative to Δ*G*_H*_. Their relationships are illustrated in Figure 5d,e. Interestingly, we observed a counterintuitive trend: the armchair edge site, which has a smaller Δ*G*_H*_, displays a smaller *ε*_d_ but larger surface DOSs; whereas, the zigzag edge site, with larger Δ*G*_H*_, exhibits a larger *ε*_d_ but smaller surface DOSs. This suggests that the conventional d‐band center theory may not be suitable for accurately evaluating the edge state HER catalytic performance in this context. In contrast, the surface DOSs display a positive correlation with the Δ*G*_H*_ for both edge sites. This behavior has previously been identified for the TSS, serving as a universal catalytic descriptor in topological nodal line materials. These findings underscore the intricate interplay between electronic structure and catalytic activity at the edge sites and challenge the conventional understanding of catalytic descriptors. This could open up new avenues for the design and optimization of 2D materials for catalysis, taking into account the unique electronic properties at the edge sites. Future studies could further explore the role of edge electronic structure in catalytic performance, potentially leading to more effective strategies for catalyst design and optimization. In addition, we have also considered the kinetics process, including the Volmer–Tafel and Volmer–Heyrovsky reaction pathways as reported for MoS_2_,^[^ ^82^, ^83^ ^]^ and obtained results are reported in Supporting Information. From the calculated energy changes between different states, we can see that the Volmer–Tafel reaction has lower activation energy compared to the Volmer–Heyrovsky reaction for both the basal plane and armchair edge. When considering the solvation effect, the overall reaction energy is only slightly affected by the presence of H_2_O. For the zigzag edge, both Volmer reactions exhibit relatively low reaction energies. However, the H_2_O solvation effect significantly deteriorates the Tafel reaction, while the Heyrovsky one remains unaffected. This additional analysis can provide a more thorough understanding of the kinetic behavior during the HER process.

## 4 Conclusion

With increasing research interest in the development of quantum topological materials for high‐performance catalytic activity in the HER, trivial topological materials have emerged as a promising research direction due to their larger availability and more robust surface states. In this context, we present a novel 2D Janus material, MoSMg, which is obtained by substituting one layer of sulfur (S) in the monolayer MoS_2_ with a layer of magnesium (Mg). The resulting monolayer MoSMg exhibits exceptional thermodynamic stability and demonstrates OAI behavior with clean OSSs. These metallic topological trivial states significantly enhance the catalytic performance of MoSMg for HER in electrochemical processes. Furthermore, we systematically investigate the catalytic performance under various hydrogen coverage conditions and our findings reveal that the OWCC site consistently displays robust HER performance, particularly at high hydrogen coverage. Additionally, we examine the catalytic activity of the edge sites in the monolayer MoSMg, considering both the armchair and zigzag edge configurations. Remarkably, we observe even more superior HER catalytic activity at the edge sites, as evidenced by the obtained near‐zero Gibbs free energies. The combination of OAI behavior and exceptional catalytic activity represents a novel finding in the field. By identifying the 2D Janus material MoSMg, our study opens up new possibilities for designing quantum electrocatalysts within the realm of trivial topological materials, with desirable performance characteristics in mind.

## Conflict of Interest

The authors declare no conflict of interest.

## Supporting information

Supplementary Material

## Data Availability

The data that support the findings of this study are available from the corresponding author upon reasonable request.
