# Supplementary material for: Unlocking Quantum Catalysis in Topological Trivial Materials: A Case Study of Janus Monolayer MoSMg
Source: Small Sci. 2024 Jul 22;4(10):2400160. doi: 10.1002/smsc.202400160 (PMC11935022; doi:10.1002/smsc.202400160)
Supplement: Supplementary file 1 — Supplementary Material [file SMSC-4-2400160-s001.pdf]

Supporting Information

Unlocking quantum catalysis in topological trivial materials: a case study of Janus monolayer MoSMg

Ying Yang, Jialin Gong, Xiaotian Wang, Zhenxiang Cheng, Tie Yang**

Table S1. The detailed atomic positions and bond length distributions of the two dimensional monolayer MoSMg.

Lattice	a (Å)	b (Å)	c (Å)
MoSMg	3.03995	3.03995	20.00000
Atom	x	y	z
Mo	0.00000	0.00000	0.49843
S	0.33333	0.66667	0.41939
Mg	0.33333	0.66667	0.61428
Bond length	$d_{S\text{-}Mo}$ (Å)		$d_{Mg\text{-}Mo}$ (Å)
MoSMg	2.36207		2.90674
Bond angle	$\theta_{Mo\text{-}Mg\text{-}Mo}$ (°)	$\theta_{S\text{-}Mo\text{-}Mg}$ (°)	$\theta_{Mo\text{-}S\text{-}Mo}$ (°)
MoSMg	63.0559	94.8657	80.1054

Table S2. The possible elementary band representation (EBR) decompositions of the two dimensional monolayer MoSMg at high symmetry points Γ, M, and K in the double space group P3m1. The first row gives the possible EBRs induced from different orbits E_i at the WPs la, lb, and lc. The other rows labeled by 1 to 15 are possible EBR decompositions. The numbers on the right are the multiplicities of each EBR in the corresponding decomposition. Three EBRs (E1@1a, E1@1b, and E1@1c) with a nonzero integer combination are indivisible and must be linked to the electron-filled WPs by 1a, 1b, and 1c.

Case	E_1E_2 @(1a)	E_1 @(1a)	E_1E_2 @(1b)	E_1 @(1b)	E_1E_2 @(1c)	E_1 @(1c)

1	0	1	0	2	4	9
2	0	1	1	4	3	7
3	0	1	2	6	2	5
4	0	1	3	8	1	3
5	0	1	4	10	0	1
6	1	3	0	2	3	7
7	1	3	1	4	2	5
8	1	3	2	6	1	3
9	1	3	3	8	0	1
10	2	5	0	2	2	5
11	2	5	1	4	1	3
12	2	5	2	6	0	1
13	3	7	0	2	1	3
14	3	7	1	4	0	1
15	4	9	0	2	0	1

Table S3. The hydrogen adsorption free energy (ΔG_{H*}) at different adsorption sites.

Adsorption Sites	ΔG_{H*} (eV)
S1	0.103
S2	0.180
S3	0.797

Table S4. The hydrogen adsorption free energy (ΔG_{H*}) at different hydrogen coverages.

Coverages	ΔG_{H*} (eV) @ S1	ΔG_{H*} (eV) @ S3
θ=1/9	0.103	0.180
θ=2/9	0.078	0.195
θ=3/9	0.141	0.208
θ=4/9	0.086	0.190
θ=5/9	0.242	0.215

Table S5. The hydrogen adsorption free energy (ΔG_{H*}) at two edge configurations.

Edge	ΔG_{H*} (eV)
zigzag	0.072
armchair	0.008

Beside the hydrogen adsorption free energy, we have also incorporated additional analysis into the kinetics of the HER process, including the Volmer-Tafel and Volmer-Heyrovsky reaction pathways as reported for MoS_2.[1, 2] Obtained results are shown in the following figures. From the calculated energy changes between different states, we can clearly observe that the Volmer-Tafel reaction has lower activation energy compared to the Volmer-Heyrovsky reaction for both the basal plane and armchair edge. When considering the solvation effect, the overall reaction energy is only slightly affected by the presence of H_2O. For the zigzag edge, both Volmer reactions exhibit relatively low reaction energies. However, the H_2O solvation effect significantly deteriorates the Tafel reaction, while the Heyrovsky one remains unaffected.

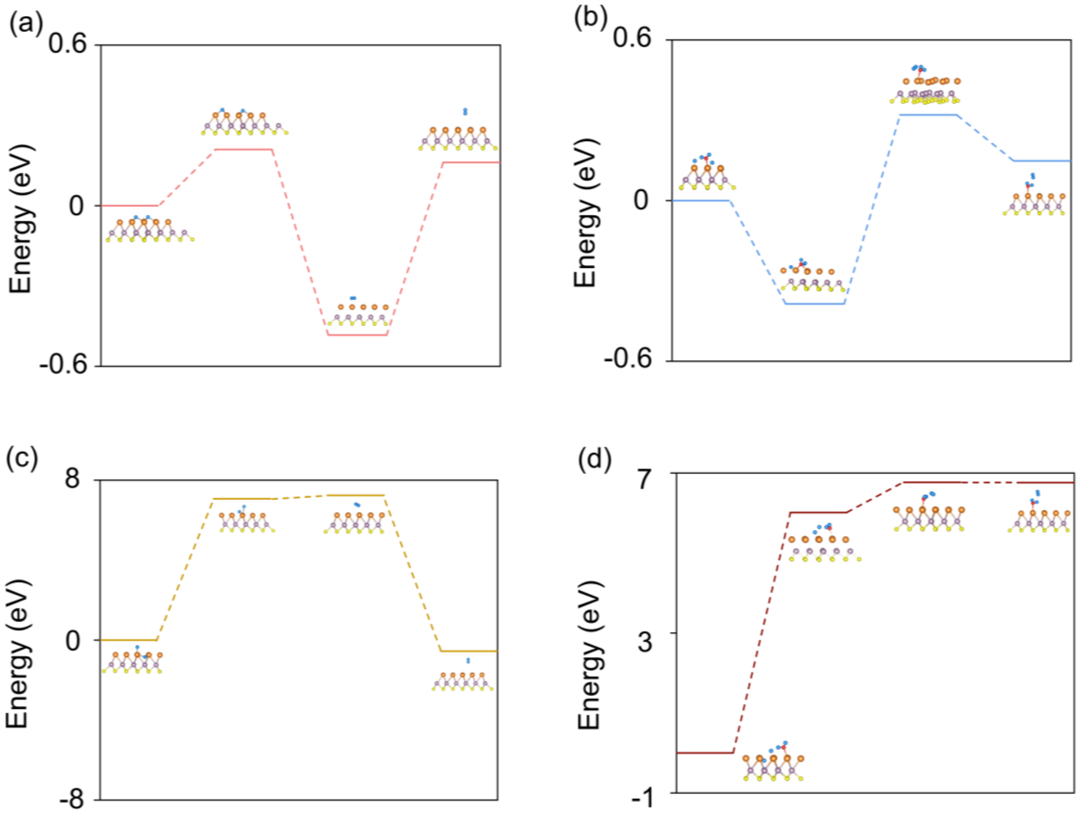

Figure S1. Geometric structures of the initial, transition and final states of the Volmer-Tafel reaction and its energetics on a) bare surface and b) in the presence of H_2O at the basal plane of the Janus monolayer MoSMg. c) and d) are results for the Volmer-Heyrovsky reaction.

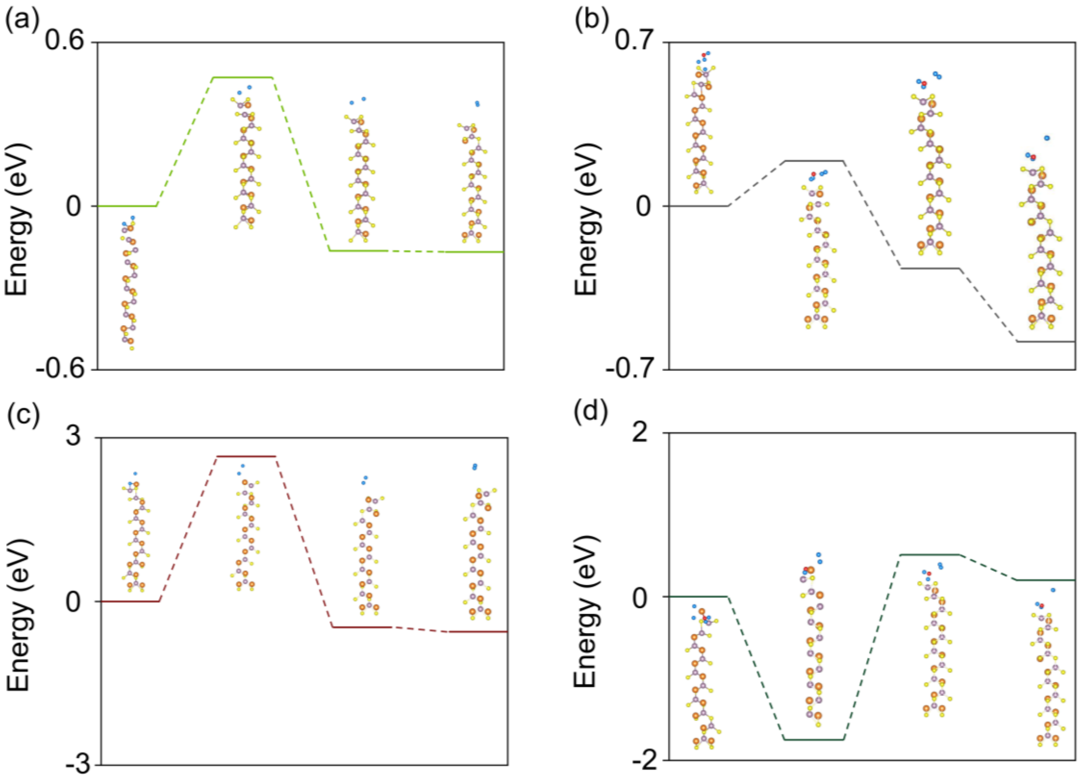

Figure S2. Geometric structures of the initial, transition and final states of the Volmer-Tafel reaction and its energetics on a) bare surface and b) in the presence of H_2O at the armchair edge of the Janus monolayer MoSMg. c) and d) are results for the Volmer-Heyrovsky reaction.

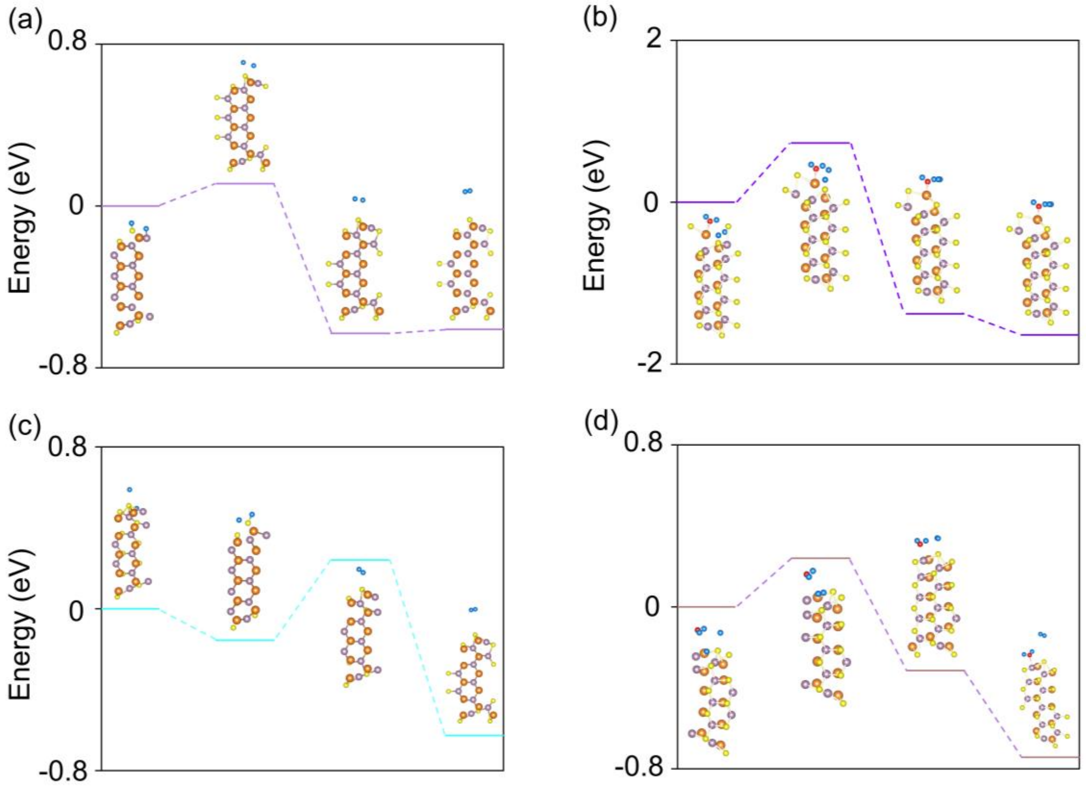

Figure S3. Geometric structures of the initial, transition and final state of the Volmer-Tafel reaction and its energetics on a) bare surface and b) in the presence of H_2O at the zigzag edge of the Janus monolayer MoSMg. c) and d) are results for the Volmer-Heyrovsky reaction.

To assess the stability of the Janus MoSMg monolayer against oxidation, we adopted a method analogous to the one used for studying surface oxidation energetics and kinetics on monolayer MoS_2.[3] In this approach, the thermodynamic stability of the oxidized surfaces is characterized by their formation energies, calculated using the following equation:

$$E^{form}(\text{ads})=E(\text{ads})-E(\text{pristine})-n\mu_O$$

where E^{form}(ads) is the formation energy of oxidized surface with total DFT energy E(ads), E(pristine) is the DFT energy of the clean surface, and μ_O is the reference chemical potential of O adsorbed species with n number of atoms. The computed value of the formation energy for the full oxygen coverage condition is -4.53 eV/O, indicating the thermodynamic stability of the oxidized surface. The negative oxidation energy suggests that the Janus MoSMg surface, similar to MoS_2, is prone to oxidation. To mitigate the oxidation of the Janus MoSMg monolayer, we propose utilizing a carbon shell protection method, which has been shown to be effective for MoS_2.[4] We anticipate that similar protective measures could be applied to the

MoSMg monolayer, potentially preserving its structural integrity and electrocatalytic performance.

References

[1] W. Li, G. Liu, J. Li, Y. Wang, L. Ricardez-Sandoval, Y. Zhang, Z. Zhang, Hydrogen evolution reaction mechanism on 2H-MoS2 electrocatalyst, Applied Surface Science, 498 (2019) 143869.
[2] N. Abidi, A. Bonduelle-Skrzypczak, S.N. Steinmann, Potential and support-dependent hydrogen evolution reaction activation energies on sulfur vacancies of MoS2 from GC-DFT, International Journal of Hydrogen Energy, 48 (2023) 8478-8488.
[3] S. KC, R.C. Longo, R.M. Wallace, K. Cho, Surface oxidation energetics and kinetics on MoS2 monolayer, J Appl Phys, 117 (2015).
[4] R. Marks, A. Schranck, R. Stillwell, K. Doudrick, Stability of 2H- and 1T-MoS2 in the presence of aqueous oxidants and its protection by a carbon shell, Rsc Adv, 10 (2020) 9324-9334.
